# Medical and Physical Intelligence

**Published:** 1806-01

**Authors:** 


*Grenville Street, Brunswick Square.*

---

### MEDICAL AND PHYSICAL INTELLIGENCE.

[ FOREIGN AND DOMESTIC. ]

---

### DR. GIRDLESTONE'S ADDRESS TO THE INHABITANTS OF YARMOUTH.

IF the prejudices against VACCINATION cannot be overcome in a few words, it is not likely that the greatest part of the Inhabitants of a Town can be convinced by any long production.

Let us examine the FOUR ARGUMENTS which are too often used against this inoculation, viz.

1*st. That it is a preventive of the small pox for only a few years.*

2*d. That the Vaccine preventive is taken from a beast.*

3*d. That to inoculate is taking the work out of the hands of the Almighty.*

4*th. That Children can never die better than young.*

THE FIRST ARGUMENT, *that the Vaccine Pox is a preventive for only a few years,* was answered by Dr. Jenner's first publication, where the names are given of persons who had been inoculated with Small Pox matter thirty and more years after they had had the Cow Pox, and could not receive the Small Pox: And all the experiments which have been since made, prove that the Cow Pox is a preventive for ever from the fatality of the Small Pox.

THE SECOND ARGUMENT, *that the Vaccine preventive is taken from a beast.* The prevention of starving is often taken from the same animal. No person objects to butter, cheese, milk, or beef, on account of the diseases incident to the Cow, which are commonly much simpler than those of the human race.

THE THIRD ARGUMENT. *That to inoculate is taking the work out of the hands of the Almighty.* I shall wave the questions which might grow out of this argument, as why good and evil are mixed in our present state of probation, or the discussion of the hacknied question about liberty and necessity; and only ask, whether the most RIGID NECESSITARIAN can acquit his conscience in permitting his Child to burn to death, or to fall into scalding scalding water, or to be run over by a Horse, or bitten by a Dog, if he had it in his power to prevent such misfortunes? and whether it is not equally his duty, if he knows the means, to protect his offspring from the fatality of diseases as from any other calamity.

THE FOURTH ARGUMENT, *that a Child can never die better than young*, may lead to a great neglect of duty: and is at best but a short sighted selfishness: for it is impossible for any Parent to tell which Child might become the comfort and support of his old age, or the Protector of the rest of his Children, Relatives, or Friends.

In recommending VACCINATION to the consideration of the Inhabitants, I can have no interest but the preservation of the Eyes, Limbs, and Lives of their offspring, for there is no disease which oftener induces the Scrofula and loss of Sight than the Small Pox, whether it takes place by the natural or the artificial means.

---

The long desired measure of restricting the Medical Profession to the hands of none but well-instructed practitioners in the country as well as in London, is at length about to be carried into effect. The provisions, as far as they concern regulars, are intended to be PROSPECTIVE, and consequently will not operate upon the present generation; but as these die away or retire, their situations will be occupied by persons of suitable and competent education. Plans of the same kind have lately been introduced into Divinity and the Law, and no good reason can be assigned for not extending the principle to Medicine, which has already done so much good in the sister-professions.—The plan has already obtained the countenance and support of many of the most respectable physicians and surgeons of the metropolis, and we anticipate a speedy and zealous co-operation of the faculty in all parts of the kingdom, in support of a measure which is eminently calculated to increase the credit of the profession, and to make its followers infinitely more useful to the community.

---

A disease of a very extraordinary nature has appeared among the labourers in a coal-mine at Anzain, near Valenciennes, the cause of which appears to be confined to a single shaft in that mine. The face and the whole body assume a very dark yellow colour, and the patient falls into a state of languor and exhaustion, in which he lingers several months, sometimes more than a year, when death generally supervenes. Four men, who had been thus affected more than eight months, were removed to the hospital of the School of Medicine at Paris. The characteristic symptoms they exhibited were, an universal discolouration, swelling, inability to walk without oppression, palpitations, and habitual perspiration. One of these poor men fell a victim to the malady. M. Halle, on opening his body, was particularly struck with the absence of blood in almost every part; and this he justly considers as as one of the most remarkable circumstances of the disease. The mode of treatment adopted with respect to the other, after this discovery, was more successful. The first indication of this favourable change appeared in the projection of the blood vessels. At the period when M. Hallé drew up the above statement, the three labourers had almost entirely recovered, and their skin had resumed nearly its natural colour.

---

The following is a method of preparing a luminous bottle, which will give sufficient light during the night to admit of the hour being easily seen on the dial of a watch : — A phial of clear white glass, of a long form, should be chosen, and some fine olive oil should be heated to ebullition in another vessel. A bit of phosphorus, the size of a pea, should be thrown into the phial, and the boiling oil carefully poured over it, till the phial is one-third filled.—The phial must now be carefully corked, and when it is to be used, it should be unstopped, to admit the external air, and closed again. The empty space of the phial will then appear luminous, and give as much light as a dull ordinary lamp.—Each time that the light disappears, on removing the stopper it will instantly reappear. In cold weather the bottle should be warmed in the hands before the stopper is removed. A phial thus prepared may be used every night for six months.

---

Mr. Francis Pachiani, professor of natural philosophy at Florence, has discovered the constituent principles of muriatic acid, which had hitherto escaped the researches of every chemist. It is an oxyd of hydrogen, perhaps at its lowest degree of oxygenation. He forms it at pleasure, and consequently the accuracy of his statement cannot be doubted.

---

*The Spring Courses of Lectures at the London Hospital.*

Practice of Physic, by Dr. Cooke.
Chemistry, by Dr. Hamilton and Dr. Yelloly.
Theory of Medicine and Materia Medica, by Dr. Frampton.
Theory and Practice of Midwifery, by Dr. Dennison.
Anatomy, Physiology, and the Principles and Operations of Surgery, by Mr. Headington and Mr. Frampton.
Anatomical Demonstrations and Dissection, by Mr. Armiger.
Surgery, by Mr. Headington.
Chemical Observations on Surgical Cases, by Sir William Blizard and Mr. T. Blizard.

Particulars may be known by applying to Mr. Price, Apothecary at the Hospital.

---

*Medical Theatre, St. Bartholomew's Hospital.*

The Spring Courses of Lectures will begin at this Theatre on Tuesday, January the 21st. On the Theory and Practice of Medicine, by Dr. Roberts and Dr. Powell. — On Anatomy and Physiology, siology, by Mr. Abernethy.—On the Theory and Practice of Surgery, by Mr. Abernethy. — On Comparative Anatomy and the Laws of organic Existence, by Mr. Macartney.— On Chemistry, by Dr. Edwards.— On Midwifery and the Diseases of Women and Children, by Dr. Thynne. —The Anatomical Demonstrations and Practical Anatomy, by Mr. Lawrence. Further particulars may be learned by applying to Mr. Nicholson, at the Apothecary's Shop, St. Bartholomew's Hospital.

---

The Croonian Lecture for the present season has been read at two of the meetings of the Royal Society by Mr. CARLISLE. The subject was, "The Power and particular Structure of the Muscles of Fishes." After several minute physiological explanations of the nature and peculiar structure of the muscles of fishes, and their invariable insertion in fleshy instead of tendinous matter; he proceeded to detail his experience on their power and particular use, in enabling the animal to move with rapidity through a fluid so dense as water. He ascertained that the muscles of the sides are solely those by means of which the fish advances: that the pectoral and abdominal fins serve only to raise or lower, and balance it in the water.

---

Dr. BADHAM's Spring Course of Lectures on Medicine and Chemistry, will be commenced on Thursday Morning the 24th of January, at eight o'clock, and will be continued at the usual hours. A Third Course will be given in the Summer, and will commence about the end of May. A Prospectus may be had.

---

Mr. BROOKES's Spring Course of Lectures, on Anatomy, Physiology, and Surgery, will commence on Monday, the 20th of January, at two o'clock in the afternoon, at the Theatre of Anatomy, Blenheim-street, Great Marlborough-street,

---

Dr. CLARKE's Lectures on Midwifry, and the Diseases of Women and Children, will in future be read only at the house of Mr. Clarke, No. 10, Upper John-street, Golden-square. A Course will begin on Thursday, January 23, and the lectures will be continued every day for the convenience of students attending the hospitals. For farther particulars enquire of Dr. Clarke, No. 1, New Burlington-street, or of Mr. Clarke, No. 10, Upper John-street, Golden-square.

---

Mr. CHEVALIER will commence his next Course of Lectures on the Principles and Operations of Surgery, on Wednesday the 29th of January, at seven o'clock in the evening, at his house in South Audley-street, Grosvenor-square.

---

Mr. MACARTNEY is about to deliver a Course of Lectures at the Medical Theatre. St. Bartholomew's Hospital, on Comparative Anatomy and the Laws of Organic Existence.

On Monday, January 13, the Course of Lectures on the Principles and Practice of Surgery, will be recommenced by Mr. JOHN PEARSON, Senior Surgeon of the Lock Hospital, and Asylum, and of the Public Dispensary.

---

Dr. REID's Spring Course of Lectures, on the Theory and Practice of Medicine, will commence on the 25th of January. Particulars may be learned by applying at Dr. Reid's house, Grenville-street, Brunswick-square; or at the Finsbury Dispensary, St. John's-square, Clerkenwell.

---

Mr. JOHN TAUNTON, Surgeon to the City and Finsbury Dispensary, &c. will commence his Spring Course of Lectures on Anatomy, Physiology, and Surgery, on the 18th of January, at his Theatre in Grenville-street.

---

Dr. TROTTER, of Newcastle, has just published "PROPOSAL FOR DESTROYING THE FIRE AND CHOAK DAMPS OF COAL MINES;" occasioned by some dreadful accidents that have lately happened in that neighbourhood. The subject has attracted a considerable share of public attention in the district; but we are sorry to learn, that some unworthy attempts have been tried to defeat the benevolent intentions of the active philosopher.

---

Dr. JOHN REID's Treatise on the Origin, Progress, Prevention, and Treatment of Consumption, will be ready for publication in a few days. In the construction of his work, Dr. R. has attempted to adapt it not to professional readers merely, but likewise to general perusal. He has endeavoured, in a particular manner, to illustrate the importance of early and carefully discriminating between the characters of true pulmonary affection and those disorders which often assume a fictitious resemblance of genuine phthisis.

---

In the course of next month will be published, the second volume of Mr. FOX's Work upon the Teeth, containing the History and Treatment of the Diseases of the Teeth, the Gums, and the alveolar processes, the operations which they require, and observations on other diseases of the mouth.

---

Dr. RUSH, of America, is arranging a complete edition of his medical works, which will be comprized in three vols. octavo.

---

Dr. HUTCHINSON, of Philadelphia, is writing a Treatise on Ulcers, particularly those of the lower extremities.

